# Fruit and Vegetable Juices as Functional Carriers for Probiotic Delivery: Microbiological, Nutritional, and Sensory Perspectives

**DOI:** 10.3390/microorganisms13061272

**Published:** 2025-05-30

**Authors:** Renata Žvirdauskienė, Vesta Jonikė, Loreta Bašinskienė, Dalia Čižeikienė

**Affiliations:** 1 Department of Food Science and Technology, Kaunas University of Technology, Radvilėnų Rd. 19, 50254 Kaunas, Lithuania; renata.zvirdauskiene@ktu.lt (R.Ž.); loreta.basinskiene@ktu.lt (L.B.); 2 Microbiology Laboratory, Institute of Agriculture, Lithuanian Research Centre for Agriculture and Forestry, Instituto al. 1, Akademija, 58344 Kedainiai, Lithuania; 3 Department of Biology, Vytautas Magnus University, Universiteto Str. 10, 53361 Akademija, Lithuania; vesta.jonike@vdu.lt

**Keywords:** probiotic fermentation, functional beverages, fruit and vegetable juice, lactic acid bacteria, non-dairy probiotic carriers, sensory properties

## Abstract

Fermenting fruit and vegetable juices with probiotic bacteria is becoming a popular way to create functional drinks, offering an alternative to traditional dairy-based probiotic products. These plant-based juices are naturally rich in nutrients that help support the growth and activity of various probiotic strains. They also meet the rising demand for lactose-free, vegan, and clean-label options. This review looks at the key microbiological, nutritional, and sensory aspects of probiotic fermentation in juice. Common probiotic groups like *Lactobacillus*, *Bifidobacterium*, *Lactococcus*, *Bacillus*, and *Streptococcus* show different abilities to adapt to juice environments, affecting properties such as antioxidant levels, shelf life, and taste. The review also explores how factors like pH, sugar levels, heating, and storage can influence fermentation results. New non-thermal processing methods that help maintain probiotic survival are also discussed. Since fermented juices can sometimes develop off-flavors, this paper looks at ways to improve their taste and overall consumer appeal. Finally, future directions are suggested, including personalized nutrition, synbiotic products, and advanced encapsulation technologies. Overall, probiotic fermentation of fruit and vegetable juices shows strong potential for developing a new generation of healthy and appealing functional foods.

## 1. Introduction

The human gut microbiota plays a fundamental role in host physiology and metabolism. Meanwhile, an increasing number of pieces of clinical evidence support the health benefits of consuming probiotic-enriched products. These benefits extend beyond improving gastrointestinal function and alleviating diarrhea or constipation; they include reducing the risk of allergies and skin conditions, managing immune-related disorders, lowering serum cholesterol levels, and even contributing to the prevention of colorectal cancer [1,2,3,4].

The past few decades have witnessed a marked rise in consumer demand for probiotic-enriched food products [5,6,7,8]. This surge has stimulated both scientific inquiry and innovation in the food industry, driving the development of functional food components and new formulations that incorporate live microbial cultures with demonstrated health benefits [9].

The global market for probiotic products is expanding rapidly and is projected to grow from USD 69.8 billion in 2024 to USD 102.1 billion by the end of 2029 [10].

The health-promoting properties of probiotics depend primarily on their concentration in food products and their ability to survive the harsh conditions of the gastrointestinal tract. Despite the growing commercial interest in probiotic strains, many currently available probiotics are sensitive to environmental stressors and tend to lose viability during processing and storage. It has been established that probiotic viability should remain at a minimum of 10^7^ CFU/mL throughout a product’s shelf life, regardless of the strain used [11,12,13,14]. Consequently, selecting an appropriate food matrix for probiotic delivery is critical.

Among the most straightforward and consumer-preferred delivery systems for probiotics are beverages, which provide an effective medium for the fermentation of probiotic bacteria and the transport of both nutritional and bioactive compounds to the host [15]. Depending on the raw materials used, probiotic beverages can be classified into dairy-based, fruit- and vegetable-based, legume-based, cereal-based, and fermented-tea- or coffee-based categories.

At the start of the 21st century, approximately 74% of probiotic foods on the market were dairy-based [16]. However, rising prevalence of lactose intolerance, the adoption of vegan diets, and increased consumer interest in cholesterol-lowering diets [7,17] have catalyzed a global search for plant-based alternatives to traditional dairy matrices.

In the last decade, fruit and vegetable juices—either individually or in blends—have gained traction as promising carriers for probiotics under gastrointestinal stress conditions. These juices provide essential nutrients that support probiotic viability [9,18,19,20,21] and are broadly acceptable across all demographic groups [21,22]. Fermented fruit and vegetable juices are rich in vitamins, minerals, and antioxidants, and the fermentation process can enhance the bioavailability of these compounds. Juices fermented using probiotics are therefore emerging as increasingly popular functional beverages [23].

**Figure 1:**
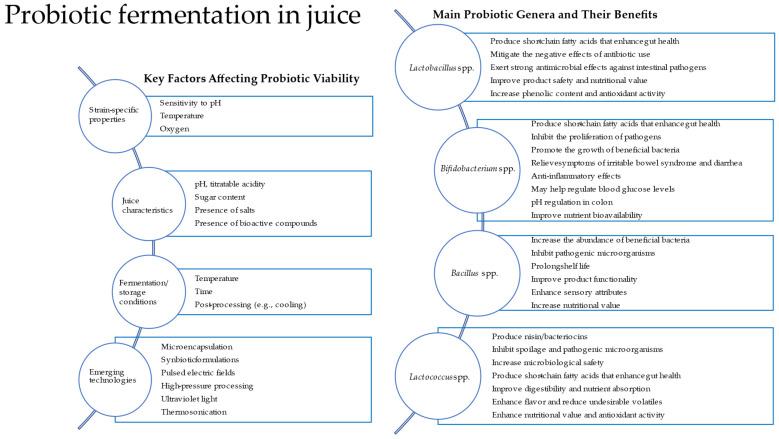
Diagram showing key factors affecting probiotic viability and the main probiotic genera used and their benefits. Emerging technologies can be applied in probiotic juice fermentation.

Traditionally, probiotics such as *Lactobacillus* and *Bifidobacterium* have been associated with fermented dairy products. However, recent research has demonstrated their viability and metabolic activity in plant-based substrates. This review summarizes the probiotic strains commonly used in the fermentation of fruit and vegetable juices, the criteria for selecting such strains, the suitability of juices as fermentation substrates, and the influence of fermentation on product characteristics and bacterial viability. A conceptual diagram (Figure 1) shows key factors affecting probiotic viability, the main probiotic genera used, and their benefits, and emerging technologies can be applied in probiotic juice fermentation.

## 2. Factors Influencing Probiotic Fermentation in Fruit and Vegetable Juices

Fruit and vegetable juices serve as nutrient-rich substrates that support the growth and metabolic activity of probiotic microorganisms. These juices are natural sources of fermentable sugars, essential vitamins, minerals, dietary fibers, and antioxidants, which collectively create a favorable environment for probiotic colonization and activity [24,25,26,27]. Furthermore, bioactive compounds such as polyphenols and flavonoids found in these matrices offer additional health benefits by mitigating chronic diseases [28]. Some juices also naturally contain prebiotics, which further enhance the viability and longevity of probiotics [29,30,31]. Notably, many fruit and berry juices exhibit antimicrobial activity against pathogenic microorganisms [32,33,34,35].

Despite their favorable composition, several interrelated factors critically influence the viability and functionality of probiotics during fermentation and subsequent storage.

- Strain-Specific Characteristics: Different probiotic strains confer distinct metabolic profiles and sensory outcomes in fermented beverages. Therefore, strain selection is a fundamental determinant of fermentation success.
- pH and Acidity: The initial and dynamic pH values of juices play a pivotal role. Many fruit juices are inherently acidic, and fermentation further lowers pH levels due to organic acid production, potentially compromising probiotic survival. Titratable acidity, water activity, and the presence of salts, sugars, and other compounds also modulate the microbial environment [36].
- Processing and Fermentation Conditions: The survival of probiotics is also affected by juice pre-treatment and fermentation parameters such as temperature, duration, and cooling rate after fermentation [37].

These factors must be carefully optimized to ensure effective fermentation and stable probiotic delivery through juice-based functional beverages.

## 3. Key Probiotic Strains Used in Juice Fermentation

The choice of probiotic strains for juice fermentation significantly influences the physicochemical and functional characteristics of the final product. Selected strains must demonstrate resilience under processing, storage, and gastrointestinal conditions, while also contributing positively to the organoleptic profile of the product [38,39,40,41].

In plant-based beverages, strain selection is guided by the ability to ferment complex carbohydrates and produce desirable sensory attributes [42]. Moreover, strains must not generate undesirable metabolites, off-flavors, or biogenic amines during fermentation [43,44,45,46,47].

The most frequently utilized probiotic microorganisms include species from the genera *Lactobacillus*, *Bifidobacterium*, *Enterococcus*, *Lactococcus*, *Streptococcus*, and heat-resistant spore-formers like *Bacillus*. Some strains from *Escherichia* and *Saccharomyces* are also explored for specific applications. Most widely used are heterofermentative lactic acid bacteria (LAB), particularly *Lactobacillus* and *Enterococcus*, along with bifidobacteria [48].

### 3.1. Application of Lactobacillus Strains in Juice Fermentation

Species within the *Lactobacillus* genus are Gram-positive, non-motile, catalase-negative, rod-shaped bacteria that are either anaerobic or facultatively anaerobic. They thrive in environments enriched with 5–10% CO_2_ and require specific nutritional components such as amino acids, peptides, fatty acids, vitamins, carbohydrates, and nucleotide derivatives. Their optimal growth temperature ranges between 30 and 40 °C, though they can proliferate in a broader range from 5 °C to 53 °C. The ideal pH for their growth lies between 5.5 and 5.8 [49].

Among the most frequently used *Lactobacillus* strains in juice fermentation are *L. plantarum*, *L. acidophilus*, and *L. casei*, all of which are valued for their robust fermentation performance and potential health benefits.

*L. acidophilus* is one of the most important strains within the *Lactobacillus* genus. It primarily colonizes the small intestine, where it helps maintain the integrity of the intestinal barrier, ensuring efficient nutrient absorption and proper digestive function. This strain is known for its ability to mitigate the negative effects of antibiotic use, resist bile acids, and exert strong antimicrobial effects against intestinal pathogens, including coliform strains of *E. coli* [50].

*L. acidophilus* remains viable at pH 2.0 for up to 2 h of incubation and tolerates bile salt concentrations of 0.3%. It can be produced on an industrial scale and remains viable and stable both in food products and within the intestinal microbiota. *L. acidophilus* meets all essential FAO/WHO criteria for classification as a probiotic bacterium [2,51].

Studies have confirmed that *Lactobacillus* strains remain viable in fermented fruit juices. For example, *L. paracasei* demonstrated promising viability during cold fermentation of chokeberry juice [52]. Other strains, including *L. rhamnosus*, *L. acidophilus*, and *L. fermentum*, have been shown to significantly increase the probiotic count in fermented juices and maintain viable counts exceeding 10⁷ CFU/mL [53,54]. *L. plantarum*-fermented pineapple juice also exhibited high probiotic viability and enhanced antioxidant properties, confirming its suitability as a non-dairy probiotic beverage [55].

Apple juice has been successfully used as a substrate for probiotic fermentation. *L. plantarum* was able to reach the viable cell concentrations required for health benefits [56]. Additionally, apple juice fermented with *L. acidophilus* and *L. plantarum* showed potential in reducing contaminants such as mycotoxins. These probiotic strains were shown to remove patulin, a common contaminant in apple juice, thereby improving product safety and nutritional value [57]. This added value further strengthens the role of probiotics, not only in fermentation, but also in ensuring food safety.

The fermentation of pear juice with various *Lactobacillus* strains increased antioxidant activity and modified phenolic compound profiles, highlighting the enhancement of health-promoting properties through fermentation [53,55,58].

Similarly, the fermentation of strawberry juice with *Lactobacillus* strains improved radical scavenging capacity, associated with increased phenolic content during fermentation [59,60]. These results underscore the beneficial impact of fermentation on juice quality and health attributes.

*Lactobacillus* strains are also known to produce short-chain fatty acids (SCFAs) during fermentation, compounds that support gut health. SCFA production helps maintain microbiome balance and reduce intestinal inflammation, reinforcing the role of fermented fruit and vegetable juices as functional foods [61,62]. Additional studies have shown that vegetable juices such as cabbage or spinach fermented with LAB may improve calcium bioavailability due to enzymatic activity [63].

### 3.2. Bacillus Genus and Its Role in Fermented Juices

The genus *Bacillus* includes Gram-positive, rod-shaped, spore-forming bacteria that are typically aerobic or facultatively anaerobic. While commonly associated with soil microbiota, certain non-pathogenic *Bacillus* species have been successfully applied as probiotics [64]. These strains are excellent candidates for use in probiotic food products, particularly due to their ability to maintain viability at elevated temperatures [65,66]. Optimal growth for *Bacillus* spp. occurs between 35 and 50 °C, with a preferred pH range of 5.5–6.5.

*Bacillus coagulans* is particularly noteworthy due to its capacity to form heat-resistant spores, making it suitable for application in high-temperature environments [64,67,68,69]. Another advantage of *B. coagulans* is its high biomass yield in bioreactor cultures, surpassing that of other probiotic strains such as *L. rhamnosus*, *L. salivarius*, *L. plantarum*, and *L. lactis* [70].

*B. coagulans* has been recognized as safe for human consumption by the U.S. Food and Drug Administration (FDA) and the European Food Safety Authority (EFSA). It is included in both the GRAS (Generally Recognized as Safe) and QPS (Qualified Presumption of Safety) lists and is currently used in numerous commercial probiotic formulations [71].

Its ability to remain viable in acidic conditions reinforces its suitability for application in probiotic juice fermentation. For instance, Ebrahimi et al. (2021) found that *B. coagulans*-enriched fruit juices increased the levels of immunoglobulins and lymphocytes in athletes [72]. Similarly, Almada-Érix et al. (2021) demonstrated that orange juice supplemented with *B. coagulans* altered the gut microbiota in Wistar rats by increasing the abundance of beneficial bacteria such as *Lactobacillus* and *Bacillus* spp. [73]. These results emphasize the potential of *Bacillus* strains to modulate gut flora in a health-promoting direction.

The fermentation of fruit and vegetable juices with *Bacillus* probiotics not only improves product functionality but also enhances sensory attributes and inhibits pathogenic microorganisms. Flavor and aroma profiles of fermented juices are often altered positively due to the metabolic activity of *Bacillus* spp. Sireswar et al. (2018) demonstrated that the synergistic effect between *Bacillus* strains and phenolic compounds in fruit juices significantly suppressed foodborne pathogens while enhancing overall flavor acceptability [74].

Further support for these findings comes from Maia et al. (2023), who showed that incorporating *Bacillus* strains in juice fermentation altered both volatile and non-volatile compound profiles [60]. This modification led to improved sensory perception and consumer appeal.

Recent literature by Saud et al. (2024) underscores that probiotic fermentation with *Bacillus* not only prolongs shelf life but also increases nutritional value by enhancing levels of beneficial compounds while inhibiting spoilage organisms [75]. These benefits position *Bacillus*-fermented juices as promising functional beverages with extended stability, enhanced safety, and superior consumer appeal.

### 3.3. Bifidobacterium Genus and Its Application in Juice Fermentation

The *Bifidobacterium* genus currently comprises over 30 recognized bacterial species, many of which are considered essential components of the human gut microbiota [76]. These bacteria produce lactic and acetic acids, contributing up to 70% of the energy required by the colonic epithelium [77]. This activity enhances the intestinal barrier function and supports the maintenance of a balanced gastrointestinal environment.

The production of lactic acid by *Bifidobacterium* strains helps maintain an optimal colonic pH, inhibiting the proliferation of pathogenic microorganisms while promoting the growth of beneficial bacteria [78]. Important species with well-documented probiotic properties include *B. lactis*, *B. bifidum*, *B. longum*, *B. infantis*, and *B. breve*. However, the abundance of bifidobacteria tends to decrease with age, stress, and poor dietary habits [79].

Among these, *B. longum*, *B. breve*, and *B. infantis* are frequently highlighted not only for their beneficial effects on health but also for their potential in fermentation processes. *B. longum* is capable of metabolizing a broad spectrum of carbohydrates, producing lactic acid and short-chain fatty acids (SCFAs) that contribute to gut health [80]. Its ability to ferment polysaccharides improves the nutritional quality of fermented juices by breaking down complex carbohydrates into simpler, more bioavailable sugars for both the host and the microbial community [81].

*B. breve* has demonstrated probiotic properties that enhance gastrointestinal health and systemic wellness. It has been associated with improved immune responses in the gut and symptom alleviation in conditions such as irritable bowel syndrome (IBS) and diarrhea [82]. SCFA production by *B. breve* is particularly important, as these metabolites contribute to the integrity of the intestinal barrier and have anti-inflammatory effects [83].

The selection of appropriate *Bifidobacterium* strains for fruit and vegetable juice fermentation significantly affects the sensory and functional properties of the final product. In a recent study by Güler et al. (2024), new *Bifidobacterium* strains isolated from infant feces were evaluated for their probiotic potential in juice matrices [84]. The selected strains exhibited favorable characteristics that could enhance the health-promoting value of fermented fruit and vegetable juices.

Juices enriched with oligosaccharides—naturally present in many fruits—offer a conducive environment for *Bifidobacterium* growth. Fermentation under these conditions not only improves probiotic activity but also results in the formation of bioactive compounds, increasing the nutritional value of the final product [85]. Moreover, the ability of *Bifidobacterium* strains to outcompete harmful intestinal pathogens contributes to both the safety and shelf life of fermented juices [82,83].

There is also evidence that the postprandial consumption of *Bifidobacterium*-fermented juices may help regulate blood glucose levels, suggesting potential applications in functional foods for diabetes management [86].

Genomic analyses of *Bifidobacterium* strains have revealed considerable diversity in metabolic profiles, which may be exploited to optimize fermentation processes for specific juice types [81,87]. These metabolic adaptations are crucial for defining the final flavor, aroma, and health benefits of the fermented product.

### 3.4. Lactococcus Genus, Health Benefits, and Functional Potential

Among the various probiotic strains applied in juice fermentation, *Lactococcus lactis* stands out for its widespread use and recognized safety. It has been granted GRAS (Generally Recognized as Safe) status and has a long history of safe use in the production of dairy products such as cheese and fermented milk [88,89].

*L. lactis* primarily metabolizes lactose into lactic acid, thereby contributing to pH reduction and inhibition of spoilage and pathogenic microorganisms. This acidification process not only preserves the structural and microbiological integrity of fermented products but also generates a range of flavor-enhancing metabolites [90,91]. Additionally, *L. lactis* is known to produce antimicrobial peptides, such as nisin and bacteriocins, which further enhance food safety and extend shelf life [90,92].

Several studies have confirmed the antimicrobial efficacy of *L. lactis* in fermented juices. For instance, Özdogan et al. (2012) and Boumaiza et al. (2018) demonstrated that *L. lactis* effectively inhibits foodborne pathogens, increasing the microbiological safety of probiotic beverages [93,94].

Beyond its antimicrobial properties, *L. lactis* contributes positively to gut health. It produces short-chain fatty acids (SCFAs), which are essential for maintaining intestinal barrier function, modulating immune responses, and reducing inflammation [95,96]. Although *L. lactis* is traditionally associated with dairy fermentation, its performance in fruit and vegetable juice fermentation has gained increasing attention. For example, Siroli et al. (2019) reported that the fermentation of carrot juice with *L. lactis* not only reduced undesirable volatile compounds but also enhanced the nutritional value of the final product [92]. The study showed that *L. lactis* fermentation reduced the levels of potentially harmful substances and improved the overall safety and acceptability of the juice.

These findings are consistent with other studies suggesting that lactic acid fermentation can neutralize antinutritional factors present in raw plant-based juices, thus improving their bioavailability and health benefits [97]. *L. lactis* also contributes to improved digestibility and nutrient absorption through the breakdown of complex carbohydrates and the release of bioactive peptides, thus providing a functional advantage to consumers looking for health-oriented choices [98].

Fermentation trials using *L. lactis* have also been extended to non-traditional plant-based substrates. Aloe vera juice, for instance, demonstrated enhanced antioxidant activity when fermented with *L. lactis* owing to the improved bioavailability of phenolic compounds [99]. This suggests that *L. lactis* not only ferments juices but also increases their health benefits. Similarly, a coconut-based beverage fermented with *L. lactis* showed probiotic functionality and enhanced nutritional characteristics [100].

These examples highlight the adaptability of *L. lactis* to a range of juice matrices, underscoring its potential as a versatile probiotic in the development of innovative plant-based functional beverages.

### 3.5. Streptococcus thermophilus: Its Comparative Performance, Limitations, and Recommendations

*Streptococcus thermophilus* is a homofermentative lactic acid bacterium best known for its central role in the production of yogurt and cheese. Most of the existing literature regarding this species pertains to its application in dairy fermentations [101]. However, due to its ability to produce exopolysaccharides (EPS) and its potential health benefits, *S. thermophilus* has attracted interest as a candidate for the development of functional non-dairy beverages [102,103].

Recent research indicates that *S. thermophilus* can be used in plant-based fermentations. It has been shown to dominate the microbial population throughout the fermentation of vegetable juices, thanks to its strong acidification ability and competitive fitness in certain substrate conditions [104,105,106]. This is because heterofermentative lactic acid cultures dominate only in the early stages of fermentation and cannot maintain their dominance throughout the process, so their dominance is quickly replaced by homofermentative ones [105,107]. However, if only homofermentative bacteria dominate the starter, the sensory properties of the product may deteriorate, an overly sour off-flavor may appear, and the texture of the product may change. It has been found that the fresh and pleasant taste and high sensory quality of kimchi are determined by the properties of heterofermentative lactic acid bacteria, and they are considered better candidates for starters than homofermentative ones [108,109]. This was confirmed by a comparative study by Cai et al. [110], which examined the fermentation of jujube juice using *S. thermophilus* and *L. plantarum*. The findings revealed that *S. thermophilus*-fermented juice had significantly poorer sensory properties compared with that fermented with *L. plantarum* [110].

Given these observations, *S. thermophilus* may not be the optimal choice for the standalone fermentation of fruit and vegetable juices, especially in formulations where sensory quality is a priority. However, it may still serve as a useful component in multi-strain probiotic mixtures or in substrates where its fast acidification and EPS production offer technological advantages, such as viscosity improvement or texture stabilization.

To achieve balanced flavor and functional outcomes, combinations of *S. thermophilus* with heterofermentative bacteria or yeasts may provide better results, particularly for consumer-acceptable probiotic beverages.

### 3.6. Use of Multi-Strain Probiotic Mixtures

Scientific evidence suggests that combining multiple probiotic strains may offer enhanced health benefits compared with single-strain applications. This is particularly relevant for combating gastrointestinal pathogens, where synergistic interactions between strains may lead to broader antimicrobial activity. For example, multi-strain probiotic formulations have been found to be more effective against pathogens such as *Escherichia coli*, *Campylobacter jejuni*, and *Shigella* [111,112].

Accordingly, mixed cultures are sometimes employed in juice fermentation to achieve improved functionality, product safety, and probiotic efficacy. Commonly used probiotic combinations include strains such as *L. acidophilus*, *L. rhamnosus* GG, *Saccharomyces boulardii*, *B. bifidum*, and *B. coagulans* [113].

#### 3.6.1. Considerations for Strain Compatibility and Technological Viability

When designing multi-strain formulations for juice fermentation, several factors, such as strain compatibility, product characteristics, temperature regime, bacteriophage risk, and raw material variability, must be taken into account. Microorganisms must not inhibit one another’s growth or function. The biochemical composition of the juice may affect probiotic stability and metabolic activity. Fermentation and storage temperatures must support the viability of all included strains. Mixed cultures may be vulnerable to phage attack, which can disrupt fermentation. Probiotic resistance to variations in juice composition is essential for consistent product quality. Mesophilic strains are more suitable for fermentation processes at 20–30 °C, while thermophilic strains are preferred for processes conducted at 40–45 °C [114].

#### 3.6.2. Safety Considerations

Although most probiotics—especially those from the *Lactobacillus* and *Bifidobacterium* genera—are considered safe due to their long history of use, strain-specific safety assessments remain essential. Some strains may pose risks if not properly evaluated, particularly in immunocompromised individuals [115,116].

The selection of multi-strain consortia for probiotic juice products must therefore be guided by rigorous safety, efficacy, and stability evaluations to ensure consumer protection and product performance.

## 4. Environmental Parameters: pH and Related Factors for Viability

The pH level of fruit and vegetable juices plays a critical role in determining the survival and stability of probiotic bacteria during fermentation and subsequent storage. These juices are naturally rich in organic acids, resulting in an inherently low pH that may create unfavorable conditions for microbial viability. Acidic environments and the associated antimicrobial effects of organic acids are considered key stressors limiting probiotic persistence in juice-based systems.

Probiotic bacteria generally prefer a pH range close to neutral. Numerous studies have demonstrated that pH changes during fermentation have a direct impact on probiotic viability [24,36,117,118,119,120]. Furthermore, although sugars in fruit juices serve as a useful energy source for probiotics, their metabolism during fermentation leads to the production of additional organic acids, thereby further decreasing the pH [55,121].

Viability losses may also occur during later stages of storage due to continued acidification, autolysis, and enzymatic degradation of dead cells [122]. This highlights the importance of not only the initial pH but also its evolution over time as a determinant of probiotic stability. Interestingly, some strains—particularly within the *Lactobacillus* genus—are better adapted to acidic conditions. These bacteria have demonstrated the ability to survive in juices with pH values ranging from 3.7 to 4.3, making them suitable candidates for acidic substrates [12].

### 4.1. Strategies to Enhance Probiotic Viability in Acidic Conditions

Several approaches have been explored to improve the survival of probiotics in low-pH environments.

### 4.2. Microencapsulation

Encapsulating probiotic cells in protective matrices has proven to be one of the most effective methods for shielding them from acidic stress. Studies have shown that encapsulated probiotics exhibit higher survival rates during fermentation and storage compared with free cells [122,123,124,125,126].

### 4.3. Sensory Acceptability

In addition to improving viability, encapsulation may also enhance the overall sensory acceptability of the product by mitigating the negative effects of acid-induced cell death and metabolite release [127].

### 4.4. Strain Selection

Selecting acid-tolerant lactic acid bacteria can significantly increase probiotic survival under acidic conditions [128].

### 4.5. Inclusion of Antioxidants

Certain antioxidant compounds have been shown to support probiotic viability, possibly by modulating the fermentation environment or reducing oxidative stress [129,130].

### 4.6. Storage Conditions

Maintaining appropriate storage temperatures is crucial. Higher storage temperatures can accelerate metabolic processes and exacerbate pH fluctuations, negatively affecting probiotic stability [126,131].

## 5. Thermal Processing of Juices

Fruit and vegetable juices are typically preserved using physical, chemical, or biological methods, all aimed at ensuring microbiological safety, shelf life, and quality. The primary objective of these processing techniques is to eliminate pathogenic microorganisms while preserving key attributes essential for both consumer health and probiotic functionality.

The effectiveness of thermal processing is influenced by several factors, including the juice’s composition, microbial diversity, and selected processing parameters. Among the most commonly used approaches prior to fermentation are thermal treatments of varying intensities.

Pasteurization remains the standard thermal treatment method for ensuring microbial safety and prolonging the shelf life of juices. This process involves the application of elevated temperatures to inactivate spoilage microorganisms and undesirable enzymes. For example, high-temperature short-time (HTST) pasteurization has been shown to effectively reduce microbial loads in various juices, but it may also degrade heat-sensitive nutrients and negatively impact sensory attributes [132,133]. Recent studies emphasize the need to balance microbial inactivation efficacy with the preservation of nutritional quality, suggesting that mild heat treatments—such as low-intensity thermal processing or thermosonication—can maintain essential quality attributes while achieving microbial reduction [134,135].

### 5.1. Emerging Thermal Technologies

Innovative thermal processing techniques such as ohmic heating and thermosonication have gained popularity due to their ability to deliver more controlled heat transfer while minimizing the degradation of bioactive compounds. Studies show that thermosonicated juices exhibit improved color, retention of bioactive compounds, and enhanced sensory characteristics compared with their conventionally pasteurized counterparts [134]. This method effectively reduces microbial populations while preserving both sensory and nutritional properties—an essential consideration for subsequent probiotic fermentation.

Moreover, as previously discussed, changes in juice pH during processing can complement thermal treatment strategies. Fruit juices, typically acidic in nature, enhance microbial inactivation due to the increased heat sensitivity of spoilage microorganisms under low-pH conditions. A combination of moderate thermal treatment and juice acidification creates a synergistic effect. Modeling studies confirm that the interplay between pH and temperature significantly influences the thermal resistance of *Alicyclobacillus acidoterrestris*, a common spoilage microorganism in fruit juices [136,137].

### 5.2. Non-Thermal Alternatives

A range of non-thermal methods have been investigated as either alternatives or complements to thermal processing. Technologies such as pulsed electric fields (PEFs), high-pressure processing (HPP), and ultraviolet (UV) light have been evaluated for their ability to inactivate microorganisms without compromising the nutritional integrity of juices [135,138]. These techniques preserve heat-sensitive micronutrients while achieving microbial safety, making them particularly attractive for probiotic juice applications [139].

The thermal treatment and stabilization of fruit and vegetable juices play a critical role in preparing substrates for probiotic fermentation. A combination of conventional thermal processing with emerging technologies—such as thermosonication or non-thermal methods—can ensure microbiological safety while preserving the sensory and nutritional quality of the juice. These attributes are essential for the successful development of health-promoting probiotic beverages.

## 6. Impact of Probiotic Fermentation on the Physicochemical and Sensory Properties of Juices

One of the key challenges in developing probiotic-enriched fruit and vegetable juices is preserving their original physicochemical and sensory characteristics. Thus, it is essential to evaluate the metabolic activity of different probiotic microorganisms within juice matrices and their capacity to alter product sensory attributes.

The application of various *Lactococcus* species in fruit and vegetable juice fermentation has yielded promising results, particularly in enhancing the sensory quality of the final products. Studies of volatile compound profiles in juices fermented with *Lactococcus lactis* demonstrate that specific strains can generate complex flavor and texture characteristics, potentially increasing consumer acceptance [140]. Metabolites produced during fermentation contribute to unique flavor profiles, making the beverages more appealing while retaining their health-promoting properties [91]. Successful fermentations of apple and grape juices with *L. lactis* have led to improved flavor, aroma, and shelf-life stability [97,141]. Additionally, fermented apple juice has shown increased concentrations of bioactive compounds and antioxidants, further enhancing its health benefits [142].

Other investigations have demonstrated that the antioxidant activity of apple juice increases after fermentation, linked to the bioconversion of phenolic compounds. This suggests that fermentation can enhance the overall nutritional profile of the juice [143]. Fermentation with *Lactiplantibacillus plantarum* has been associated with high esterase activity, significantly influencing aroma development, while maintaining probiotic viability above recommended levels [144].

Recent studies also indicate that fermentation with *Lactobacillus* strains can intensify juice color and the concentration of antioxidant compounds, yielding probiotic beverages that are both nutritionally valuable and visually appealing [59,145]. These findings support earlier results by Hossain et al. (2020), which showed that LAB fermentation improves both the biochemical and sensory qualities of fruit juices, enhancing their health value and market potential [55].

Tomato juice has likewise shown substantial improvement through fermentation with lactic acid bacteria (LAB), including increased probiotic counts and enhanced antioxidant activity [146,147]. Fermentation with different *Lactobacillus* strains improved both the antioxidant profile and the sensory acceptability of tomato juice [148,149]. *L. acidophilus* was reported to induce substantial flavor modifications and prolong shelf life due to its pronounced acidification capabilities [150]. Similarly, *L. plantarum* strains isolated from tomatoes improved the sensory profile and probiotic attributes of fermented tomato juice, while also reducing pH levels [151].

Pumpkin juice fermentation resulted in desirable sensory properties and sustained high levels of probiotic viability [152]. Carrot juice has been identified as a promising delivery medium for probiotics, yielding a naturally sweet flavor that enhances consumer acceptance [153].

Fermentation of carrot and beetroot juices has also proven suitable for the growth of *L. plantarum*, *L. acidophilus*, and *L. casei*. During fermentation, these substrates exhibited reduced sugar content and pH values alongside increased acidity—indicators of a successful metabolic activity that promoted probiotic growth [119]. The properties of beetroot juice further support its efficacy as a fermentation substrate, offering favorable flavor and nutritional benefits [43,154]. The probiotic fermentation of beetroot juice not only ensured excellent bacterial viability but also conferred antimicrobial properties to the final beverage [43].

In the case of pomegranate juice, despite its initially low pH (~3.09), LAB such as *L. plantarum* were able to thrive, indicating high acid tolerance—an essential trait for successful fermentation in acidic matrices [155]. Fermentation improved the antioxidant activity and modified sugar and organic acid profiles, thereby enhancing the health-promoting characteristics of the final product [25].

Pineapple juice fermented with various LAB strains also exhibited improved antioxidant capacity [156], reinforcing the idea that sugar-rich juices are excellent substrates for supporting probiotic viability and metabolic activity. Studies have identified *L. casei*, *L. rhamnosus*, *L. paracasei*, and *L. reuteri* as particularly well suited to fermenting pineapple juice [45].

Multiple studies have confirmed that most probiotics can successfully grow in fruit and vegetable juices or their blends [28,56,117,131,155,156,157,158,159,160,161,162,163,164,165]. Typically, the fermentation process increases lactic acid concentrations in juice, improving both flavor and nutritional value. A summary of probiotic strains used in juice fermentation and their advantages and limitations is provided in Table 1.

## 7. Future Perspectives and Conclusions

The regulation of non-dairy probiotics varies significantly across regions, reflecting diverse approaches to food safety, public health, and market regulation. These regulatory differences have a substantial influence on the development and commercialization of probiotic enriched products.

In Europe, probiotics are subject to stringent rules shaped by both EU and member state-specific legislation, particularly to comply with the EU Nutrition and Health Claims Regulation (NHCR) [166]. In the United States, the Food and Drug Administration (FDA) oversees the use of the term “probiotic” and its associated health claims. However, regulatory overlaps with pharmaceutical laws can sometimes lead to the misclassification of food products, thereby affecting product development and public understanding [167].

In Southeast Asia, regulations are tailored to local markets and cultural contexts, with specific definitions and guidelines for probiotic products [168]. These varying frameworks influence consumer behavior, especially when considered alongside regional dietary practices. Moreover, the complexity of regulatory environments may hinder market entry for new products and contribute to reduced consumer confidence.

Despite these challenges, interest in probiotic-rich functional foods is growing around the world, as more people recognize the link between diet, gut health, and overall well-being. Fruit and vegetable juices are becoming a strong alternative to dairy-based probiotic products, especially for those with lactose intolerance, dairy allergies, or those following vegan or cholesterol-conscious diets. As research moves forward, fermented plant-based drinks are being seen as multifunctional systems—not only supporting probiotic survival but also delivering health-promoting compounds with antioxidant, anti-inflammatory, and antimicrobial effects.

Studies show that a wide range of probiotic species—especially from the *Lactobacillus*, *Bifidobacterium*, *Lactococcus*, *Streptococcus*, and *Bacillus* genera—can successfully ferment fruit and vegetable juices. These probiotics survive and stay active in acidic juice environments, while also improving the nutritional value, taste, and safety of the final product. Fermentation usually boosts the levels of beneficial compounds like organic acids, phenolics, and antioxidants, which support the health benefits of the beverage.

However, some challenges remain before these products can be widely commercialized. Key issues include keeping probiotics alive during storage, avoiding unwanted changes in taste, coping with low pH conditions, and ensuring each strain remains safe and effective. Success in this field depends on carefully choosing the right juice bases, fermentation cultures, and processing methods—including how the product is treated and preserved after fermentation.

Looking into future perspectives, new screening techniques and genomic tools can help identify strong, acid-resistant probiotic strains that are well suited for specific juice types. Additionally, protective technologies like microencapsulation can help maintain probiotic viability during storage and ensure they reach the gut alive. Mixing probiotics with prebiotics (to form synbiotics), plant polyphenols, or other health-boosting compounds may improve both the health effects and the taste of the product. Innovative methods like high-pressure processing (HPP), pulsed electric fields (PEFs), and thermosonication may improve safety while preserving sensitive nutrients and live probiotics. Research that combines sensory science, consumer studies, and nutrition can help develop probiotic drinks that are both effective and enjoyable for a wide range of people.

Integrating probiotics into fruit and vegetable juice matrices provides a new and versatile platform to deliver health-promoting microorganisms to diverse consumer groups. While dairy-based functional products have long dominated the probiotic market, non-dairy alternatives are increasingly gaining consumer interest due to their natural bioactive compounds, broad demographic compatibility, and favorable regulatory perceptions.

In conclusion, the development of probiotic-fermented fruit and vegetable juices is a future-oriented and sustainable direction for functional food innovation. With continued scientific advances in microbiology, food technology, and systems biology, it is expected that the next generation of probiotic beverages will not only meet consumer expectations for taste and well-being, but also contribute to the global shift towards personalized nutrition and preventive healthcare.

## Figures and Tables

**Table 1:** Summary of probiotic strains used in juice fermentation: advantages and limitations.

Probiotic Strains	Juices Used in Fermentation	Main Advantages	Main Limitations	References
*Lactobacillus* spp. (*L. plantarum*, *L. acidophilus*, *L. casei*, *L. rhamnosus*, *L. paracasei*, *L. fermentum*, *L. reuteri*)	Apple, aronia, beetroot, carrot, pear, pineapple, pomegranate, pumpkin, strawberry, tomato	High viability in juice matrices Enhance antioxidant activity SCFA production Detoxify patulin Improve biochemical and sensory juice properties	pH sensitivity for some strains Need tailored fermentation conditions	[53,54,55,56,57,59,119,150,155]
*Lactiplantibacillus plantarum*	Apple, aronia, beetroot, carrot, grape, pineapple, pomegranate, tomato	Excellent acid tolerance Boosts esterase activity and aroma Strong metabolic activity Maintains high LAB counts after fermentation	Oxidative sensitivity in long-term storage	[55,56,110,144,151]
*Bacillus* spp. (*B. coagulans*)	Orange, mixed fruit/vegetable	Spore-forming and acid-tolerant Enhance immunity and gut microbiota Inhibit pathogens Increase shelf life	Safety and sensory effects need control Spore germination risk	[60,72,73,74,75]
*Bifidobacterium* spp. (*B. longum*, *B. breve*, *B. infantis*, *B. bifidum*, *B. lactis*)	Fruit and vegetable juices enriched with oligosaccharides (e.g., apple, berry—assumed, pear, tropical)	SCFA production with anti-inflammatory effects Enhance gut barrier and regulate colonic pH Improve nutrient bioavailability and inhibit pathogens Improve sensory and functional quality of fermented juices	Oxygen-sensitive, viability affected by pH, temperature Fermentation behavior varies across strains	[65,67,69,72,73]
*Lactococcus lactis*	Aloe, apple, carrot, coconut, grape	SCFA production Improves taste and aroma Improves gut health Reduces antinutritional factors Enhances nutrient bioavailability	Limited juice use compared with dairy Sensory effects strain dependent	[92,97,99,100,141]
*Streptococcus thermophilus*	Jujube	SCFA production	Inferior flavor vs. *L. plantarum* Narrow substrate applicability	[110]
*Leuconostoc* spp. (heterofermentative LABs)	Cabbage, spinach, vegetable	Enhance traditional fermented flavors (e.g., kimchi) Complex aroma profile	Less explored in direct juice fermentations	[106,108]
General LAB (Lactic Acid Bacteria)	Beetroot, cabbage, carrot, grape, pineapple, spinach tomato	Boost antioxidants and shelf life Versatile across many matrices Support probiotic viability	Function is highly strain-specific Monitoring fermentation dynamics critical	[63,145,146,147,156]

## Data Availability

No new data were created or analyzed in this study.
